# A Fair Individualized Polysocial Risk Score for Identifying Increased Social Risk in Type 2 Diabetes

**DOI:** 10.21203/rs.3.rs-3684698/v1

**Published:** 2023-12-06

**Authors:** Yu Huang, Jingchuan Guo, William T Donahoo, Zhengkang Fan, Ying Lu, Wei-Han Chen, Huilin Tang, Lori Bilello, Aaron A Saguil, Eric Rosenberg, Elizabeth A Shenkman, Jiang Bian

**Affiliations:** 1 Department of Health Outcomes and Biomedical Informatics, University of Florida, Gainesville, FL, USA; 2 Pharmaceutical Outcomes & Policy, University of Florida, Gainesville, FL, USA; 3 Division of Endocrinology, Diabetes and Metabolism, University of Florida College of Medicine; 4 Department of Medicine, University of Florida College of Medicine; 5 Department of Community Health and Family Medicine, University of Florida College of Medicine; 6 Division of General Internal Medicine, Department of Medicine, University of Florida College of Medicine

**Keywords:** Machine learning, Type 2 diabetes, Machine Learning, Prediction, Fairness

## Abstract

**Background:**

Racial and ethnic minority groups and individuals facing social disadvantages, which often stem from their social determinants of health (SDoH), bear a disproportionate burden of type 2 diabetes (T2D) and its complications. It is crucial to implement effective social risk management strategies at the point of care.

**Objective:**

To develop an electronic health records (EHR)-based machine learning (ML) analytical pipeline to address unmet social needs associated with hospitalization risk in patients with T2D.

**Methods:**

We identified real-world patients with T2D from the EHR data from University of Florida (UF) Health Integrated Data Repository (IDR), incorporating both contextual SDoH (e.g., neighborhood deprivation) and individual-level SDoH (e.g., housing instability). The 2015–2020 data were used for training and validation and 2021–2022 data for independent testing. We developed a machine learning analytic pipeline, namely individualized polysocial risk score (iPsRS), to identify high social risk associated with hospitalizations in T2D patients, along with explainable AI (XAI) and fairness optimization.

**Results:**

The study cohort included 10,192 real-world patients with T2D, with a mean age of 59 years and 58% female. Of the cohort, 50% were non-Hispanic White, 39% were non-Hispanic Black, 6% were Hispanic, and 5% were other races/ethnicities. Our iPsRS, including both contextual and individual-level SDoH as input factors, achieved a C statistic of 0.72 in predicting 1-year hospitalization after fairness optimization across racial and ethnic groups. The iPsRS showed excellent utility for capturing individuals at high hospitalization risk because of SDoH, that is, the actual 1-year hospitalization rate in the top 5% of iPsRS was 28.1%, ~13 times as high as the bottom decile (2.2% for 1-year hospitalization rate).

**Conclusion:**

Our ML pipeline iPsRS can fairly and accurately screen for patients who have increased social risk leading to hospitalization in real word patients with T2D.

## Introduction

Diabetes affects 529 million people worldwide and the number is projected to more than double in the next three decades, reaching 1.3 billion by 2050.^1^ Over 90% of diabetes cases are type 2 diabetes (T2D).^2^ Existing research has shown that social determinants of health (SDoH)—”*the conditions in the environments where people are born, live, learn, work, play, worship, and age,*”^3,4^ such as education, income, and access to healthy food, play a critical role affecting a wide range of health outcomes, including the development and prognosis of T2D.^5–7^ Moreover, health disparities in T2D have been widely documented over the past decades.^8–10^ Racial and ethnic minority groups and individuals experiencing social disadvantages—often rooted in their SDoH—bear a disproportionate burden of T2D and its complications.^11–13^ As such, diabetes is a public crisis that must be managed with sensitivity to patients’ unmet social needs to improve T2D outcomes and health equity.

The US health care system has begun embracing the need to address patients’ social needs, including screening for SDoH at the point of care. For example, the Centers for Medicare & Medicaid Services (CMS) have made proposals to require SDoH screening (e.g., housing stability, food insecurity, and access to transportation) in annual beneficiary health risk assessments. Despite this push, only 16%-24% of clinics and hospitals provide SDoH screening,^15^ and the actual utilization rate is very low.^16^ In a national network of community health centers, only 2% of patients were screened for SDoH, and most had only one SDoH documented.^17^ The reasons for the extremely low rate of SDoH screening are multiple. ^18^ First, existing screening tools are not automated, making them difficult to adapt to clinical workflows. ^19,20^ In addition, almost all tools were developed for universal screening but were not validated to predict specific conditions and outcomes such as diabetes.^21–23^ Furthermore, screening for individual SDoH items at the point of care is not only inefficient, increasing the provider documentation burden, but also inadequate given the known complex interplay among the SDoH.^24–27^ *Figueroa et al*. called for using a Polysocial Risk Score (PsRS) approach,^28^ yet existing PsRS studies include only individual-level SDoH examined in small cohort studies with limited generalizability.^29–31^ It is essential to consider *both* contextual (e.g., neighborhood deprivation) and individual-level SDoH (e.g., if the individual has instable housing) in one model given their known *interactions*, especially for T2D, as shown by us and others.^24,25,27,32^

The increasing availability of real-world data (RWD)^33,34^—such as electronic health records (EHRs) and administrative claims —and the rapid advancement of artificial intelligence (AI), especially machine learning (ML) techniques to analyze RWD, provides an opportunity to develop novel personalized tools and generate real-world evidence for improving not only health outcomes but also health equity by addressing contextual-level and individual-level SDoH. However, key data and methodologic barriers exist. For example, RWD lack integration with contextual or individual-level SDoH data. Moreover, most studies that used ML models for clinical applications^35^ did not carefully consider the inherent biases in observational RWD, such as data bias where patients of low socioeconomic status may not be well-represented in EHRs due to their limited access to healthcare.^36^ A ML model naively trained on such RWD may deliver unfair outputs for racial-ethnic minority groups and socioeconomically disadvantaged individuals^36^, leading to increased health disparities and inequity. Moreover, the black box nature of ML models limits their adoption in clinical and health care applications; and explainable AI (XAI) techniques play a significant role in bridging the gap between complex ML models and human understanding.^37–39^ Shapley Additive exPlanations (SHAP)^40^ is an increasingly used, simple tool for teasing out the contribution of individual factors to a predictive model, nevertheless, it has a limited ability to explain how factors collectively affect an outcome, given the complex interactions among factors, such as complex interplay among individual-level and contextual-level SDoH. Causal structure learning methods such as the classic PC algorithm^41^ can learn causal relationships among the factors in the format of a directed acyclic graph (DAG) from observational data, and reveal how these risk factors interact to influence outcomes, offering valuable insights into the underlying processes that drive the predictions.

Therefore, in this study, we aimed to develop an EHR-based ML pipeline, namely iPsRS, for determining if increased social risk can predict hospitalization in T2D, with in-depth consideration of model fairness and explainability. Specifically, we used RWD from the University of Florida Health (UF Health) EHRs and incorporated both individual-level and contextual-level SDoH for the iPsRS development, optimized its fairness across racial-ethnic groups, and identified key causal factors that can be targeted for interventions. With these algorithms, our long-term goal is to develop an EHR-based individualized social risk management platform that can integrate social risk management into clinical care, leading to a necessary paradigm shift in US healthcare delivery.

## Methods

### Study design and population

**Figure 1:**
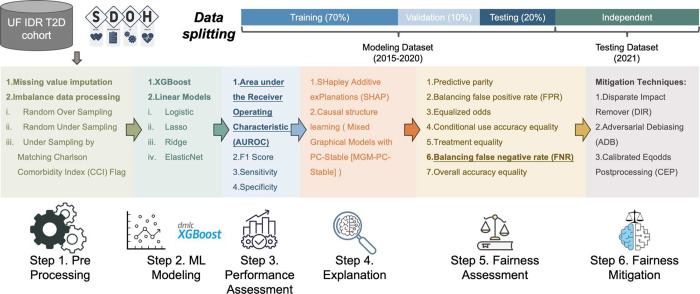
Processing workflow of the University of Florida integrated data repository type 2 diabetes cohort and the patient timeline.

We conducted a retrospective cohort study using 2015–2021 EHR data from the UF Health Integrated Data Repository (IDR), an enterprise data warehouse integrating different patient information systems across the UF Health system. UF Health provides care to more than 1 million patients with over 3 million inpatient and outpatient visits each year with hospitals in Gainesville (Alachua County), Jacksonville (Duval County), and satellite clinics in other Florida counties. In the current study, we included patients who were (1) aged 18 and older, (2) had a T2D diagnosis, identified as having at least one inpatient or outpatient T2D diagnosis (using ICD-9 codes 250.×0 or 250. ×2, or ICD-10 code E11) and ≥ 1 glucose-lowering drug prescription in (a case finding algorithm previously validated in EHRs with a positive predictive value [PPV] >94%)^42^, and (3) had at least one encounter during both baseline period and the follow up year. The index date was defined as the first recorded T2D diagnosis in the UF Health IDR data. We traced back 3 years prior to the index date as the baseline period to collect predictor information and followed up for 1 year to collect outcome (i.e., hospitalization) information (Figure 1).

### Study outcome

The study outcome was all-cause hospitalization within 1 year after the index date, identified using the first occurrence of an inpatient encounter during the follow-up year (Figure 1).

### Covariates

#### Demographics and clinical characteristics

We collected patient demographic (age, sex, and race-ethnicity) and clinical information (comorbidities, co-medications, lab values and clinical observations) for the baseline period. Race-ethnicity included four categories, including non-Hispanic White (NHW), non-Hispanic Black (NHB), Hispanic, and 5% were other races/ethnicities. The zip codes of patient residences were collected during the baseline period for contextual-level SDoH linkage.

#### Individual-level SDoH via natural language processing

We employed a natural language processing ^43,44^ pipeline that was developed by our group ^45^ to extract individual-level SDoH information from clinical notes in the baseline period, including education level (i.e., college or above, high school or lower, and unknown), employment (i.e., employed, unemployed, retired or disabled, and unknown), financial constraints (i.e., has financial constraints and unknown), housing stability (i.e., homeless or shelter, stable housing, and unknown), food security (i.e., having food insecurity and unknown), marital status (i.e., single, married or has partner, widow or divorced, and unknown), smoking status (i.e., ever smokers, never, and unknown), alcohol use (i.e., yes, no, and unknown), and drug abuse (i.e., yes, no and unknown). We also obtained insurance information (i.e., private insurance, Medicare, Medicaid, No-pay, unknown and others) from structured data.

#### Contextual-level SDoH through spatiotemporal linkage with the external exposome data

To obtain the contextual-level SDoH, we extracted the built and social environment measures (n=114 variables) including information on food access, walkability, vacant land, neighborhood disadvantage, social capital, and crime and safety, from six well-validated sources with different spatiotemporal scales (**Supplement Table S1)** built upon our prior work.^46,47^ We spatiotemporally linked these measures to each patient based on their baseline residential address (i.e., patients’ 9-digit zip codes). Area-weighted averages were first calculated using a 250-mile buffer around the centroid of each 9-digit ZIP code. Time-weighted averages were then calculated, accounting for each individual’s residential address.

## Development of ML pipeline for iPsRS

**Figure 2:**
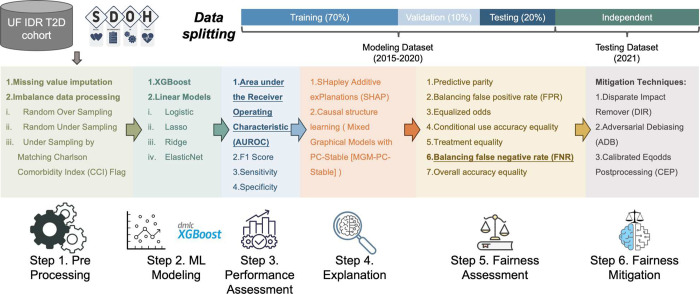
Data analytics pipeline.

Figure 2 shows our overall analytics pipeline. First, we imputed missing data and then adopted balance processing techniques (Step 1. Preprocessing). After that, we trained a set of machine learning models by using grid search cross-validation to identify the best hyperparameters (Step 2. ML Modeling). Next, we evaluated the model prediction performance (Step 3. Performance Assessment) and utilized XAI and causal structure learning techniques to identify important causal SDoH contributing to the hospitalization outcome (Step 4. Explanation). Finally, we assessed the algorithmic fairness (Step 5. Fairness Assessment) and implemented a range of fairness mitigation algorithms to address the identified bias (Step 6. Fairness Mitigation).

### Data preprocessing

We imputed missing values using the “unknown” label for categorical variables and the mean for continuous variables. Next, we proceeded to create dummy variables for the categorical variables and applied min-max normalization to the continuous variables.

### Machine learning model development for iPsRS

We developed the iPsRS model for predicting hospitalizations in patients with T2D using three sets of input features: (1) individual-level SDoH only, (2) contextual-level SDoH only, and (3) individual- and contextual-level SDoH combined. Two classes of commonly used ML approaches, linear and tree-based models, were employed. For the linear models, we included a range of hyperparameters and penalty functions that can be utilized in constructing different models, including logistic regression^48^, lasso regression^49^, ridge regression^50^, and ElasticNet^51^. For the tree-based models, we selected Extreme Gradient Boosting (XGBoost), which is widely recognized as one of the best-in-class algorithms for decision-tree-based models and has shown remarkable prediction performance in a wide range of studies^52–57^. Following ML best practices, the study data set was split into a modeling set that includes 2015 to 2020 data, and an independent testing set that covers data in 2021. In the modeling set, we further split the samples into training, validation, and testing sets with a ratio of 7:1:2. A five-fold cross-validation grid search was executed on the training set to optimize the model parameters, and early stopping was adopted and performed on the validation set to avoid overfitting. We employed random over-sampling (ROS), random under-sampling (RUS), and under-sampling by matching on Charlson Comorbidity Index (CCI) to address data imbalance before model training. The performance of each model was evaluated by area under the receiver operating characteristic curve (AUROC), F1 score, precision, recall, and specificity.

We acquired and assigned a hospitalization risk score using the iPsRS for each patient. We then divided the ranked risk scores into 11 risk groups (top 1–5^th^ percentile, top 6–10^th^ percentile, and following deciles), enabling us to examine the one-year hospitalization rate by risk group.^58^

### Explainable AI and causal estimates

We first utilized SHAP^40^ – a commonly used XAI technique – to identify important SDoH features contributing to iPsRS predicting hospitalizations in T2D patients. Further, we used a causal structure learning model – the Mixed Graphical Models with PC-Stable (MGM-PC-Stable)^41,59–61^ – to learn causal structures in directed acyclic graph (DAG) format explaining the potential causal relationships on how collectively the identified important SDoH features impact the hospitalization outcome in T2D patients.

### Algorithmic fairness optimization

To assess the model fairness of iPsRS, we adopted seven popular algorithmic fairness metrics,^36,62^ including predictive parity, predictive equality (false positive rate [FPR] balance), equalized odds, conditional use accuracy equality, treatment equality, equality of opportunity (false negative rate [FNR] balance), and overall accuracy equality, detailed in **Supplement S1**. We primarily focused on balancing the FNR (those whom the model deemed low risk but indeed are at high risk) across racial-ethnic groups, particularly NHB and Hispanic vs. NHW, because hospitalization is an adverse health outcome. In terms of fairness, we wanted to ensure iPsRS did not have higher FNR in the disadvantaged groups (i.e., Hispanic and NHB groups) compared to the reference group (i.e., NHW). As there is no universally accepted cut-off value of fairness, we considered the parity measure of 0.80–1.25 as statistically fair and highlighted values outside this range.^63^

Decreasing the FNR of iPsRS means minimizing the false negative errors (i.e., those whom the model deemed low risk but indeed are at high risk) in the early detection of social risks that can lead to hospitalization. We then employed different bias mitigation techniques to optimize the algorithmic fairness of iPsRS, including pre-process (Disparate Impact Remover^64^ [DIR]), in-process (Adversarial Debiasing^65^ [ADB]), and post-process (Calibrated Equalized Odds Postprocessing^66^ [CEP]) approaches. We goal was to identify the final model with a good balance between prediction utility and fairness.

Python version 3.7 with the Python libraries Sciki-learn^67^, Imbalanced-learn^68^, and statsmodels^69^ were used for data processing, modeling, and result analysis tasks, AI Fairness 360^70^ for model fairness mitigation tasks, and Tetrad^71^ for causal structure learning.

## Results

### Descriptive statistics of the study cohort

Our final analysis comprised 10,192 eligible T2D patients in the cohort. Table 1 highlights the demographics, individual-level SDoH, and key contextual-level SDoH of the study population by race-ethnicity. The mean age was 58 (± 13) years, and 58% were women. Of the cohort, 50% were NHW, 39% were NHB, 6% were Hispanic, and 5% were other races/ethnicities; 41% were enrolled in Medicare, 15% in Medicaid, 31% in private insurance, and 5.7% were uninsured. Compared with NHW patients, NHB patients were younger (54.6 vs. 58.5 years, p < 0.01) and more likely to be covered by Medicaid (41% vs. 28%, p < 0.01). We identified that 20.8% of patients were single, 58.5% were married or in a relationship, and 20.1% were widowed or divorced. Crime rates were lower in neighborhoods predominantly NHW than neighborhoods with higher diversity.

### iPsRS prediction model of hospitalizations in T2D patients.

**Figure 3:**
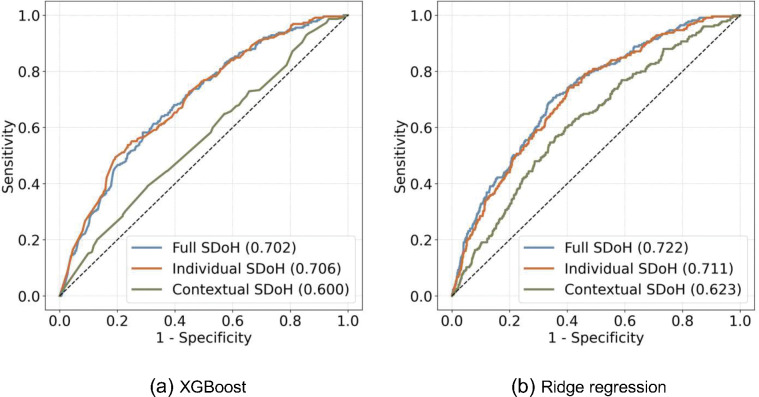
Model performance assessment of XGBoost and ridge regression.

The best-performing models generated by XGBoost and ridge regression with three different sets of SDoH (individual-level SDoH only, contextual-level SDoH only and both combined) are shown in Figure 3. The models including individual-level SDoH only had reasonably good prediction utility (AUC 0.70–0.71) and adding contextual-level SDoH modestly improved the model performance (AUC 0.72), while contextual-level SDoH by themselves had suboptimal predicting performance (AUC 0.60–0.62).

**Figure 4:**
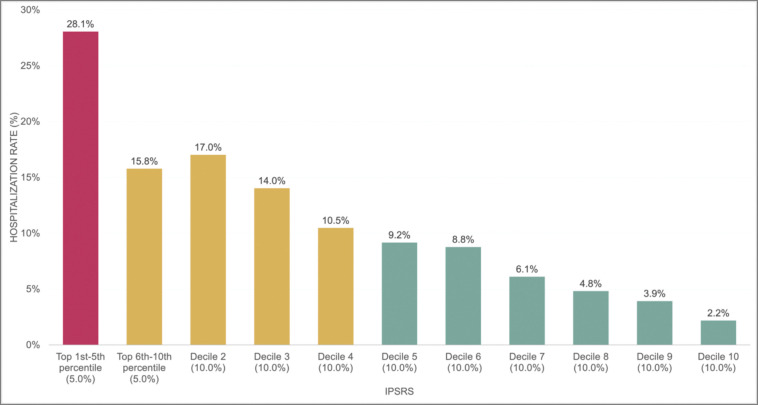
One-year hospitalization risk by iPsRS decile.

In the independent testing set (the 2021 data), we calculated the one-year hospitalization rates by decile of the XGBoost-generated iPsRS, showing an excellent utility for capturing individuals at high hospitalization risk due to SDoH (i.e., one-year hospitalization risk in the top 5% of iPsRS was 28.1%, ~13 times higher than the bottom decile, Figure 4). In a multiple logistic regression model, after adjusting for patients’ demographics and clinical characteristics, iPsRS explained 33.8% of the risk of 1-year hospitalization, per decile increase of the iPsRS, the hospitalization risk increased by 22% (adjusted odds ratio=1.22, 95%CI 1.15–1.29).

### Explainable AI to identify important SDoH contributing to iPsRS predicting hospitalization in T2D patients

**Figure 5:**
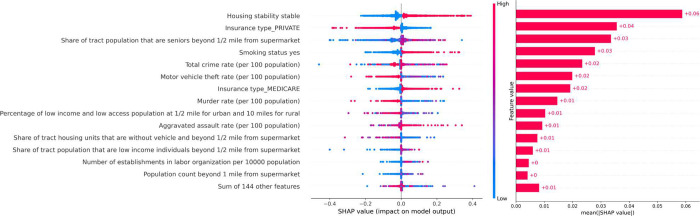
Feature importance analysis with SHAP values. SHAP values from the original XGBoost. We removed the features with an “unknown” category.

XGBoost (Figure 5) and Ridge model (**Supplement S1**) identified similar important features ranked by SHAP values. Housing stability status emerged as the most predictive feature in both models, followed by insurance type, share of tract population that are seniors beyond ½ mile from supermarket (food desert areas), and smoking status.

**Figure 6:**
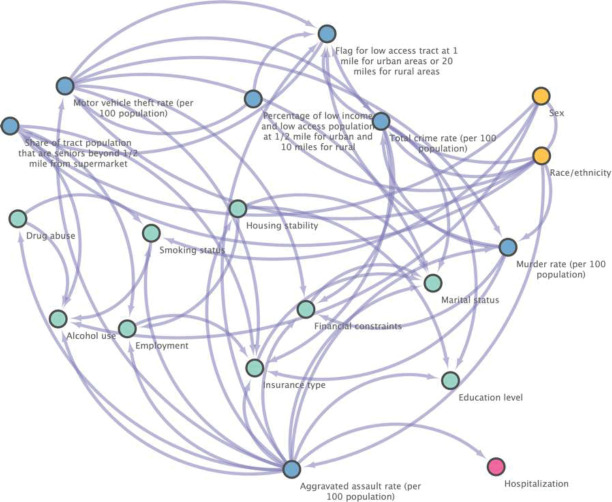
Causal graph generated by MGM-PC-Stable in the independent testing set. The yellow nodes present demographics, blue nodes stand for contextual-level SDoH and green nodes mean the individual-level SDoH, and the pink node indicates the outcome.

Figure 6 displays our exploratory analysis with causal structure learning, applying MGM-PC-Stable method to build the causal DAGs of the key SDoH (i.e., 18 unique SDoH features by combining the top-15 features from both the XGBoost and ridge regression models), resulting in a causal graph with 19 nodes (i.e., 18 SDoH and the outcome) and 36 directed edges. We identified that the aggravated assault rate in the communities where patients live is closely, causally related to the hospitalization outcome (i.e., with having a direct causal connection to hospitalization in the DAG). Furthermore, the community’s rate of aggravated assault can be viewed as a common cause of both housing stability and hospitalization, forming a fork structure where housing stability and hospitalization are dependent and correlated but conditionally independent given the aggravated assault rate. This finding aligns with the insights derived from SHAP values obtained from both XGBoost and rigid leaner models, which suggests that an individual-level SDoH, housing stability, plays a significant role in T2D hospitalization, but this influence is conditioned by the contextual-level SDoH, specifically the rate of aggravated assault in our case.

### Fairness assessment and mitigation

**Figure 7:**
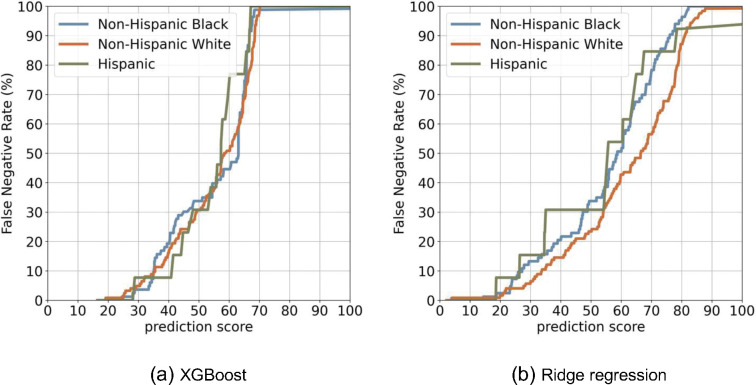
False negative rate (FNR) curve between different populations.

Figure 7 displays the FNR curves across the racial-ethnic groups, where XGBoost (Figure 7-a) appears to be fairer than the linear model (Figure 7-b). The linear model shows a greater NHB and Hispanic groups than NHW (Table 2), suggesting the model is biased against NHB and Hispanic groups compared to NHW. The overall assessment of all seven-fairness metrics can be found in **Supplement (Table S4).**

**Figure 8:**
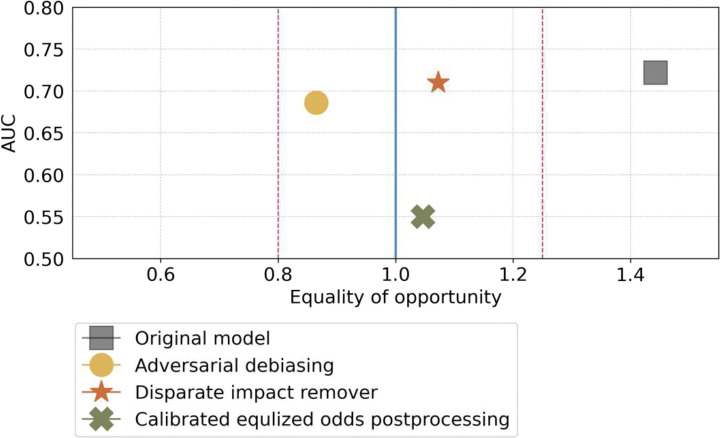

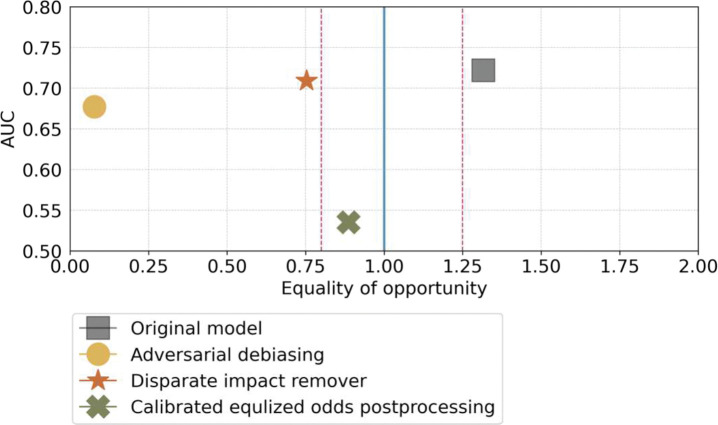
NHB (protected group) vs. NHW (privileged group) and Hispanic vs. NHW, respectively. The ideally fair line is represented by the blue line, while the range of statistically fair is shown by the red dots. the ridge regression model initially fell outside the range of statistically fair but became fairer when we employed the fairness mitigation methods CEP, DIR, and ADB, resulting in equal opportunity regarding FNR raito. **(a)** Mitigation results on the NHB vs NHW. CEP had the best fairness mitigation ability but led to a drastic decrease in model performance from 0.7220 to 0.5501, measured by AUROC, which is unacceptable. DIR and ADB resulted in an acceptable decrease in prediction performance, particularly with DIR’s AUROC decreasing from 0.7220 to 0.7100. **(b)** Mitigation results on the Hispanic vs NHW. DIR and ADB struggled to handle the fairness mitigation. These methods turned to favoritism towards the protected group (Hispanic), resulting in biased predictions for the NHW group.

Figure 8 shows the improvement status of fairness of the ridge model after employing the different bias mitigation techniques. Overall, DIR demonstrated an excellent balancing prediction utility (AUCROC=0.71 vs. 0.72 of the original model) and fairness (FNR ratio decreased from xx to 1.07) between the NHB vs. NHW.

## Discussion

In this project, we developed a fair, explainable ML pipeline, namely iPsRS, for identifying how social risk impacts hospitalizations in patients with T2D. We used UF Health EHR data, including 10,192 real-world patients with T2D, and incorporated both individual-level and contextual-level SDoH. Our results demonstrated that iPSRS is a promising tool for accurately and fairly detecting patients with a higher social risk for poor outcomes, providing explainable information on focal targets for future interventions.

Addressing patients’ unmet social needs in health care settings is a complex task due to 1) the insufficient SDoH records in EHRs (e.g., lack of use of Z codes for SDoH-associated diagnosis,^72^ and extremely low utilization of existing SDoH screening surveys embedded in EHRs^17^), 2) the concerns about the extra burden on providers^11,73,74^ and potential harms on patients^20,22,23,75^, 3) the potential data bias associated with SDoH that exists within subpopulations (e.g., racial and ethnic minority groups^12^), and 4) the observational natural of real-world EHR data (e.g., confounding and selection bias).^76^ Our EHR-based iPsRS pipeline was carefully designed to overcome the abovementioned limitations. For example, our iPsRS considers both contextual SDoH (by spatiotemporally linking patients’ EHR with the external exposome data using residential histories^32^) and individual-level SDoH (via extracting from clinical notes using our established NLP pipeline^45^). Our analyses suggested that adding contextual SDoH improved the prediction of hospitalization risk in T2D compared to the individual-level SDoH-only prediction. In addition, we employed ML approaches in EHR data to develop the iPsRS that can be embedded in EHR systems and automated for applications to minimize the extra burden of health care providers. Moreover, our model is designed to generate an initial iPsRS based on historical EHR data at the beginning of a medical encounter to guide targeted, in-person conversations between the patient and provider to collect additional SDoH information and update the iPsRS as needed, which has been carefully considered for its integration into existing clinical workflow to avoid potential harms to patients imposed by survey-type SDoH screenings and to promote patient-provider shared decision making on addressing patients’ unmet social needs. ^20,22,23,75^

With applications of multiple XAI and causal learning techniques. e.g., SHAP ^40^ values to identify key predictors and causal structure learning ^41,59–61^ to identify causal pathways, our iPsRS is able to generate interpretable outputs and has shown its ability to identify potential focal targets for intervention and policy programs to address patients’ unmet social needs essential to their health outcomes. Specifically, our SHAP value and causal structure learning model consistently identified housing instability as one of the key, modifiable factors contributing to the increased risk of hospitalization in patients with T2D. These results demonstrate a real-world use case of our iPsRS that can be used to identify SDoH-based interventions tailored to individual patients’ needs.

Another strength of our study is that we assessed the algorithmic fairness of the iPsRS and mitigated the identified bias to ensure equitable prediction across racial/ethnic groups and other sensitive attributes (i.e., sex). After fairness assessment, we identified that the ridge regression model is biased against racial and ethnic minority groups. Its prediction produced a higher FNR for both NHB and Hispanic groups compared to the NHW group, that is, NHB and Hispanic individuals who were truly at high risk of hospitalizations are more likely to be misclassified as low risk, thus more likely to miss the subsequent intervention opportunities. We applied pre-processing (DIR), in-processing (ADB), and post-processing (CEP) methods to comprehensively evaluate the effect approach to optimize iPsRS fairness. In our final model, after applying the DIR approach for bias mitigation, the iPsRS achieved an excellent prediction utility-fairness balance. That is, the AUROC was comparable (0.71 vs. 0.72 of the original model), and equal opportunity of FNR between the NHB and NHW much improved (e.g., FNR ratio decreased from 1.44 to 1.07).

We consider our PsRS pipeline has important clinical implications. Our model showed an excellent utility for capturing individuals at high hospitalization risk due to SDoH (i.e., one-year hospitalization risk in the top 5% of iPsRS was 28.1%, approximately13 times higher than the bottom decile). Our iPsRS explained 33.8% of the risk of 1-year hospitalization after adjusting for patients’ demographics and clinical characteristics, suggesting that 33.8% of increased hospitalization risk in T2D can be attributed to patients’ unmet social needs, and factors outside patients’ clinical profile. The current US health care system faces critical barriers to addressing patients’ social risks essential to health.^77^ Existing SDoH screening tools and interventions have limited efficiency and effectiveness for improving health outcomes and health equity as most of them are not tailored to address specific conditions and outcomes (e.g., T2D), and there is insufficient evidence on effective SDoH interventions, leading to a dearth of actionable knowledge (e.g., *which SDoH should be addressed and prioritized among which individuals and their effects on T2D outcomes and disparities).* RWD and AI/ML offer the opportunity to develop innovative, digital approaches to integrate social risk management into T2D care and promote a learning health community. In this project, we addressed critical methodologic barriers, including shortcomings in existing RWD infrastructure for studying SDoH, and the need for an iPsRS approach for accurate, efficient, fair, and explainable social risk screening. With these algorithms, our next step is to co-design with diverse stakeholders an EHR-based individualized social risk management platform that can integrate social risk management into clinical care, leading to a necessary paradigm shift in US healthcare delivery. This tool also provides a method of consolidating multiple components of assessing SDoH into a single, comparable score which would likely increase the likelihood of utilization by clinicians at the point of care.

Our study is subject to several limitations. First, the analysis conducted in our study was based on a cohort of patients with T2D in the state of Florida. This limited geographical scope may impact the generalizability of our findings to populations from other regions. However, our real-world T2D patients from Florida were highly diverse (e.g., 39% of Black individuals) with a mixture of rural and urban populations, reflecting the demographic changes occurring across the US. Nevertheless, future research should aim to broaden the generalizability of our iPsRS through federated learning and data from different geographic regions.^78^ Second, to ensure the automated feature, we only integrated individual-level SDoH variables that were already included in the NLP extracting SDoH pipeline (SODA^45^) and thus some of the important diabetes-related factors were missing, such as stress. We will continue developing NLP pipelines for expanding the list of SDoH extraction and updating our iPsRS model. Third, we based on ML practices to select and tune the proposed iPsRS, hence the searching space of models and hyperparameters is constrained. We plan to utilize AutoML pipelines to enhance model accuracy and reliability, while simultaneously minimizing the time and resources required to develop the next-generation model.

In this project, we developed an ML-based analytic pipeline, namely iPsRS, for identifying the increased social risk of hospitalizations in real-world patients with T2D. Our iPsRS has been shown as a promising tool to accurately and fairly identify patients’ unmet social needs essential to adverse health outcomes. The iPsRS have the great potential to be integrated into EHR systems and clinical workflow and eventually augment current screening programs for SDoH to provide physicians with an efficient and effective tool to address SDoH in clinical settings.

## Figures and Tables

**Table 1:** Summary of demographic, individual-level SDoH, and key contextual-level SDoH of the study population.

	Overall (n=10192)	NHW (n=5133)	NHB (n=4011)	Hispanics (n=495)	Others (n=553)	p-value
**Age**	58.45	60.19	56.39	55.95	59.42	0.0049
**Sex**						0.0018
*Male*	4267 (41.9%)	2470 (48.1%)	1330 (33.2%)	212 (42.8%)	255 (46.1%)	
*Female*	5925(58.1%)	2663 (51.9%)	2681 (66.8%)	283 (57.2%)	298 (53.9%)	
**Race/ethnicity**						<0.001
*NHB*	4011 (39.4%)	0 (0.0%)	4011 (100.0%)	0 (0.0%)	0 (0.0%)	
*NHW*	5133 (50.4%)	5133 (100.0%)	0 (0.0%)	0 (0.0%)	0 (0.0%)	
*Hispanics*	495 (4.9%)	0 (0.0%)	0 (0.0%)	495 (100.0%)	0 (0.0%)	
*Others*	553 (5.4%)	0 (0.0%)	0 (0.0%)	0 (0.0%)	553 (100.0%)	
**Insurance type**						<0.001
*Medicare*	4183 (41.0%)	2214 (43.1%)	1610 (40.1%)	170 (34.3%)	189 (34.2%)	
*Private*	3169 (31.1%)	1663 (32.4%)	1144 (28.5%)	148 (29.9%)	214 (38.7%)	
*Medicaid*	1511 (14.8%)	558 (10.9%)	804 (20.0%)	97 (19.6%)	52 (9.4%)	
*Nopay*	579 (5.7%)	228 (4.4%)	285 (7.1%)	38 (7.7%)	28 (5.1%)	
*Unknown*	537 (5.3%)	362 (7.1%)	84 (2.1%)	32 (6.5%)	59 (10.7%)	
*Others*	213 (2.1%)	108 (2.1%)	84 (2.1%)	10 (2.0%)	11 (2.0%)	
**Marites status**						<0.001
*Single*	2116 (20.8%)	743 (14.5%)	1221 (30.4%)	80 (16.2%)	72 (13.0%)	
*Married or has partner*	3570(35.0%)	2073 (40.4%)	1069 (26.7%)	179(36.2%)	249 (45.0%)	
*Widow or divorced*	2050 (20.1%)	888 (17.3%)	1052 (26.2%)	65 (13.1%)	45 (8.1%)	
*Unknown*	2456 (24.1%)	1429 (27.8%)	669 (16.7%)	171 (34.5%)	187 (33.8%)	
**Smoking status**						<0.001
*Ever smokers*	4096 (40.2%)	2331 (45.4%)	1473 (36.7%)	149 (30.1%)	143 (25.9%)	
*Never*	5588 (54.8%)	2525 (49.2%)	2380 (59.3%)	321 (64.8%)	362 (65.5%)	
*Unknown*	508(5.0%)	277(5.4%)	158 (3.9%)	25(5.1%)	48(8.7%)	
**Alcohol use**						<0.001
*Yes*	2631 (25.8%)	1381 (26.9%)	1012 (25.2%)	123 (24.8%)	115 (20.8%)	
*No*	6650(65.2%)	3223(62.8%)	2737(68.2%)	325 (65.7%)	365 (66.0%)	
*Unknown*	911 (9.0%)	529 (10.3%)	262 (6.5%)	47(9.5%)	73(13.2%)	
**Drug abuse**						<0.001
*Yes*	500 (4.9%)	225 (4.4%)	253 (6.3%)	16 (3.2%)	6 (1.1%)	
*No*	8487 (83.3%)	4218 (82.2%)	3409 (85.0%)	417 (84.2%)	443 (80.1%)	
*Unknown*	1205 (11.8%)	690 (13.4%)	349 (8.7%)	62(12.5%)	104 (18.8%)	
**Education level**						<0.001
*College or above*	**978 (9.6%)**	**518 (10.1%)**	**376 (9.4%)**	38 (7.7%)	46 (8.3%)	
*High school or lower*	**1110 (10.9%)**	**461 (9.0%)**	**563 (14.0%)**	50 (10.1%)	36 (6.5%)	
*Unknown*	**8104 (79.5%)**	**4154 (80.9%)**	**3072 (76.6%)**	407 (82.2%)	471 (85.2%)	
**Employment**						<0.001
*Employed*	3996 (39.2%)	2078 (40.5%)	1489 (37.1%)	207 (41.8%)	222(40.1%)	
*Unemployed*	1439 (14.1%)	570 (11.1%)	760 (18.9%)	57 (11.5%)	52 (9.4%)	
*Retired or disabled*	1948 (19.1%)	1017 (19.8%)	782 (19.5%)	68 (13.7%)	81 (14.6%)	
*Unknown*	2809(27.6%)	1468 (28.6%)	980 (24.4%)	163 (32.9%)	198 (35.8%)	
**Housing stability**						<0.001
*Homeless or shelter*	80 (0.8%)	32 (0.6%)	44 (1.1%)	3 (0.6%)	1 (0.2%)	
*Stable housing*	4215 (41.4%)	1971 (38.4%)	1933 (48.2%)	160 (32.3%)	151 (27.3%)	
*Unknown*	5897 (57.9%)	3130 (61%)	2034 (50.7%)	332 (67.1%)	401 (72.5%)	
**Food security**						<0.001
*Having food insecurity*	7052(69.2%)	3416 (66.5%)	2982 (74.3%)	300 (60.6%)	354 (64.0%)	
*Unknown*	3140 (30.8%)	1717 (33.5%)	1029 (25.7%)	195 (39.4%)	199 (36.0%)	
**Financial constraints**						0.0092
*Has financial constraints*	5172 (50.7%)	2386 (46.5%)	2323 (57.9%)	216 (43.6%)	247 (44.7%)	
*Unknown*	5020(49.3%)	2747 (53.5%)	1688 (42.1%)	279(56.4%)	306 (55.3%)	
**Percentage of low income and low access population at 1/2 mile for urban and 10 miles for rural**	0.2625 (0.1965)	0.1944 (0.1733)	0.3528 (0.1946)	0.2579 (0.1740)	0.2442 (0.1685)	0.1708
**Share of tract population that are seniors beyond 1/2 mile from supermarket**	−0.1661 (0.0949)	−0.1635 (0.1035)	−0.1669 (0.0831)	−0.1734 (0.0837)	−0.1779 (0.1000)	< 0.001
**Murder rate (per 100 population)**	0.0075 (0.0043)	0.0064 (0.0040)	0.0089 (0.0041)	0.0076 (0.0041)	0.0074 (0.0044)	< 0.001
**Aggravated assault rate (per 100 population)**	0.3867 (0.1365)	0.3767 (0.1704)	0.3980 (0.0753)	0.3994 (0.1489)	0.3858 (0.1060)	< 0.001
**Motor vehicle theft rate (per 100 population)**	0.2348 (0.0882)	0.2042 (0.0921)	0.2718 (0.0684)	0.2420 (0.0785)	0.2440 (0.0794)	< 0.001
**Flag for low access tract at 1 mile for urban areas or 20 miles for rural areas counts**						< 0.001
*Yes*	4630 (45.4%)	2091 (40.7%)	2031 (50.6%)	253 (51.1.%)	306 (55.3%)	
*No*	5562 (54.6%)	3042 (59.3%)	1980 (49.4%)	242 (48.9%)	247 (44.7%)	

**Table 2:** Statistical parity (equal opportunity) by different models on various feature sets.

Black & White	Full SDoH	Individual-level	Contextual-level SDoH
Xgboost	1.03	0.98	1.24
Ridge regression	1.44	1.18	1.45
Hispanic & White	Full SDoH	Individual-level	Contextual-level SDoH
Xgboost	1.22	1.00	1.63
Ridge regression	1.32	1.73	2.12

## Data Availability

The data presented in this study are available on request from the corresponding author. The data are not publicly available due to privacy restrictions.
